# *Bivalent Formation 1*, a plant-conserved gene, encodes an OmpH/coiled-coil motif-containing protein required for meiotic recombination in rice

**DOI:** 10.1093/jxb/erx077

**Published:** 2017-03-24

**Authors:** Lian Zhou, Jingluan Han, Yuanling Chen, Yingxiang Wang, Yao-Guang Liu

**Affiliations:** 1 State Key Laboratory for Conservation and Utilization of Subtropical Agro-Bioresources, 510642 Guangzhou, China; 2 Key Laboratory of Plant Functional Genomics and Biotechnology of Guangdong Provincial Higher Education Institutions, 510642 Guangzhou, China; 3 College of Life Sciences, South China Agricultural University, 510642 Guangzhou, China; 4 State Key Laboratory of Genetic Engineering, Institute of Plant Biology, School of Life Sciences, Fudan University, 200438 Shanghai, China

**Keywords:** Bivalent, coiled-coil motif, double strand break formation, meiosis, OmpH domain, rice.

## Abstract

Meiosis is essential for eukaryotic sexual reproduction and plant fertility. In comparison with over 80 meiotic genes identified in Arabidopsis, there are only ~30 meiotic genes characterized in rice (*Oryza sativa* L.). Many genes involved in the regulation of meiotic progression remain to be determined. In this study, we identified a sterile rice mutant and cloned a new meiotic gene, *OsBVF1* (*Bivalent Formation 1*) by map-based cloning. Molecular genetics and cytological approaches were carried out to address the function of *OsBVF1* in meiosis. Phylogenetic analyses were used to study the evolution of OsBVF1 and its homologs in plant species. Here we showed that the *bvf1* male meiocytes were defective in formation of meiotic double strand break, thereby resulting in a failure of bivalent formation in diakinesis and unequal chromosome segregation in anaphase I. The causal gene, *OsBVF1*, encodes a unique OmpH/coiled-coil motif-containing protein and its homologs are highly conserved in the plant kingdom and seem to be a single-copy gene in the majority of plant species. Our study demonstrates that OsBVF1 is a novel plant-conserved factor involved in meiotic recombination in rice, providing a new insight into understanding of meiotic progression regulation.

## Highlight

*OsBVF1* is a novel gene that is required for meiotic recombination in the monocot rice.

## Introduction

Meiosis is a specialized form of cell division that halves the chromosome number of diploid cells in producing haploid cells; it is highly conserved for sexual reproduction in most eukaryotes (Gerton and Hawley, 2005; Ramesh *et al.*, 2005). It comprises two rounds of cell division, meiosis I and meiosis II, and each round can be divided into four stages: prophase, metaphase, anaphase, and telophase. Prophase I is a relatively long phase taking up 85–95% of the total time of meiosis, and has been further divided into five stages: leptotene, zygotene, pachytene, diplotene, and diakinesis (Wang *et al.*, 2014*b*). Homologous chromosome (homolog) interaction is the crucial event during meiotic prophase I, including pairing, synapsis, recombination, and segregation. Proper interaction not only ensures the subsequently accurate segregation between homologs, but also redistributes the genetic alleles among the progeny, which has a great impact in biological diversity.

In the last three decades, molecular genetic studies have identified many genes involved in different meiotic processes in a variety of model species, such as *Saccharomyces cerevisiae*, *Drosophila melanogaster*, *Caenorhabditis elegans*, and several higher plants (Zickler and Kleckner, 1998, 2015, 2016; Osman *et al.*, 2011; Ma *et al.*, 2014; Mercier *et al.*, 2015). In the dicot model plant Arabidopsis, so far, more than 80 meiosis-related genes have been identified (Osman *et al.*, 2011; Wang *et al.*, 2014*b*; Mercier *et al.*, 2015). By contrast, only ~30 meiotic genes in the monocot model plant rice (*Oryza sativa* L.) have been cloned and functionally studied (Luo *et al.*, 2014). For example, *OsMEL1/2* and *OsAM1* are required for the initial meiotic events and their mutations cause the failure of meiotic entrance or arrest at an early stage (Nonomura *et al.*, 2007; Che *et al.*, 2011; Nonomura *et al.*, 2011). It has been well studied that meiotic recombination is initiated by the programmed formation of double strand breaks (DSBs) catalysed by SPO11, which is an evolutionarily conserved type II topoisomerase in eukaryotes (Keeney *et al.*, 1997; Grelon *et al.*, 2001; Stacey *et al.*, 2006; Yu *et al.*, 2010; An *et al.*, 2011). In rice, two SPO11 homologs, OsSPO11-1 and OsSPO11-4, were identified as being required for DSB formation (Nonomura *et al.*, 2004*a*; Yu *et al.*, 2010; An *et al.*, 2011). In addition, in yeast, there are at least eight genes involved in this process (Keeney, 2008). In Arabidopsis, AtPRD1/2/3, AtDFO, AtPCH2, and MTOPVIB were required for DSB formation (De Muyt *et al.*, 2007; De Muyt *et al.*, 2009; Zhang *et al.*, 2012; Lambing *et al.*, 2015; Vrielynck *et al.*, 2016). By contrast, only OsPAIR1, OsCRC1, OsSDS, and OsMTOPVIB were characterized as being DSB formation related in rice (Nonomura *et al.*, 2004*a*; Miao *et al.*, 2013; Wu *et al.*, 2015; Fu *et al.*, 2016; Xue *et al.*, 2016). It seems that divergence of regulation of meiotic progression exists between rice and Arabidopsis.

After DSB formation, further resection of a single end produces 3′ end overhang, which is protected by replication protein A (RPAs) proteins (Iftode *et al.*, 1999). Three RPA proteins were discovered to have a role in meiotic recombination in rice (Chang *et al.*, 2009; Li *et al.*, 2013). Further single end invasion is facilitated by RecA homologs; several rice RecA members were identified, such as OsDMC1, OsRAD51, OsRAD51C, and OsXRCC3 (Ding *et al.*, 2001; Deng and Wang, 2007; Rajanikant *et al.*, 2008; Tang *et al.*, 2014; Zhang *et al.*, 2015), suggesting that this process is conserved. As a consequence, repair of DSBs yields crossovers (COs) or noncrossovers (NCOs). Most organisms have two types of COs, of which the interference-sensitive CO (class I) depends on ZMM proteins, while the interference-insensitive CO (class II) is MUS81 dependent (Hollingsworth and Brill, 2004). In rice, several ZMM proteins such as OsMSH4, OsMSH5, OsMER3, OsHEI10, and OsZIP4 are involved in the class I CO pathway (Wang *et al.*, 2009; Shen *et al.*, 2012; Wang *et al.*, 2012*a*; Luo *et al.*, 2013; Zhang *et al.*, 2014), but the MUS81 homolog has not yet been characterized. In addition, several proteins required for meiotic chromosome segregation have been isolated in rice, such as OsSGO1 (Wang *et al.*, 2011*b*), OsREC8 (Shao *et al.*, 2011), and OsBRK1 (Wang *et al.*, 2012*b*).

The synaptonemal complex (SC) forms between homologous chromosomes and is important for the maturation of some recombination intermediates by stabilizing the paired chromosomes (Page and Hawley, 2004; Zickler and Kleckner, 2015, 2016). The SC is a tripartite structure consisting of two parallel lateral elements and a central element. The rice PAIR2 and PAIR3 are axial elements, while OsCRC1 and OsZEP1, the homolog of ZIP1 in *Saccharomyces cerevisiae* and ZYP1 in Arabidopsis, are central elements of the SC (Sym *et al.*, 1993; Wang *et al.*, 2010; Wang *et al.*, 2011*a*; Higgins *et al.*, 2005; Nonomura *et al.*, 2007; Yuan *et al.*, 2009; Miao *et al.*, 2013). Interestingly, unlike other species, partial loss of function of the rice ZEP1 has a distinct role in increase of COs (Wang *et al.*, 2010; Wang *et al.*, 2015), suggesting that different plant species may have the specific factors controlling meiosis.

In this study, we identified a sterile rice mutant with meiotic defects and isolated a gene (named *Bivalent Formation 1*, *OsBVF1*) by map-based cloning that encodes a conserved protein with a putative coiled-coil motif and an outer membrane protein H (OmpH) motif. In the *bvf1* mutant, meiotic DSB formation failed to be detected, thereby resulting in the failure of synapsis. At diakinesis, unlike the wild type (WT) that formed 12 bivalents, *bvf1* produced 24 univalents and had improper chromosome segregation in both anaphase I and II. Further analysis showed that installation of the central element, OsZEP1, of the SC was also defective. Taken together, our results reveal a new protein that is required for meiotic DSB formation and the subsequence synapsis and recombination in rice.

## Materials and methods

### Experimental materials

The *bvf1* mutant was identified from the *japanica* cv Nipponbare (Nip) mutant library induced by ^60^Co γ-ray radiation in our laboratory. The mapping populations were constructed by crossing the heterozygote (*BVF1/bvf1*) with *indica* cv Huanghuazhan (HHZ), and backcrossed with HHZ. All the materials were planted in fields in Guangzhou from spring to autumn (two growth seasons). For the recombinant screening, germinated seeds were planted in 96-well plates, and 3-week-old seedlings were used for high-throughput DNA preparation as described previously (Wang *et al.*, 2013). Detected recombinant plants were planted in field or buckets.

### Observation of pollen viability

Spikelets with mature pollen at the heading stage were collected and fixed in 70% ethanol. Then pollen grains were dissected out of anthers in 1% I_2_–KI solution. The strained pollen grains were firstly observed under a microscope (Olympus CX31), and then pictures were taken under an Axio Observer Z1 fluorescence microscope (Zeiss, Oberkochen, Germany).

### Observation of meiotic chromosome morphology

Young panicles (4–8 cm in length) of both WT and *bvf1* mutant were collected and fixed in Carnoy’s solution (ethanol:glacial acetic acid (v:v) 3:1) at room temperature in less than 24 h (Cheng, 2013). The fixed panicles were washed with 70% ethanol three to five times until the glacial acetic acid faded and then stored in it at 4 ^o^C. Pollen mother cells (PMCs) undergoing meiosis was squashed in water or phosphate-buffered saline (PBS). The slides with PMCs were then moved to a hot block at 45 ^o^C, mixing the cells with a few drops of 65% glacial acetic acid and heating for 1 min. Before the drop dried, previously frozen Carnoy’s solution was added to the center of the drop to separate the cells (Wang *et al.*, 2014*a*). After the liquid dried, 4,6-diamidino-2-phenylindole (DAPI) in anti-fade solution (Vector Laboratories, Burlingame, CA, USA) was added to the slide and covered up for observation. Chromosome images were captured under the Axio Observer Z1 fluorescence microscope.

### Expression vector construction

Total RNA from spikelets of WT rice were extracted. Total RNA (1 μg) was reverse transcribed by using M-MLV Reverse Transcriptase (Promega, Madison, WI, USA) with Oligo-T (18) as primer, the products of which were taken as the template used afterwards. The ORF sequence of *Os05g0251400* was amplified by primers pOX-*BVF1*-F/R (see Supplementary Table S1 at *JXB* online) and ligated into a binary vector so as the ORF was under the control of the ubiquitin promoter. The green fluorescent protein (GFP) fusion vectors were constructed with the Ω-PCR procedure (Chen *et al.*, 2013) with primers GFP-BVF1/BVF1-GFP. The fluorescence images were captured using an LSM 7 DUO Confocal Microscope (Zeiss).

### Rice transformation and genotyping

By *Agrobacterium* (stain EHA105)-mediated transformation, the vector constructs were transferred into callus induced from seeds of heterozygous mutant plants. Positive transformants were screened by PCR amplification with *HPT* primers and vector-specific primer pOX-T (see Supplementary Table S1 at *JXB* online), respectively. The endogenous genotypes of the transformants were identified by a semi-nested PCR with specific primers BVF1(F)/(R)/(R2) (Supplementary Table S1).

### Immunostaining assays

The methods of material fixation and slide preparation are given in Cheng (2013). After removing the coverslip, the slides were marked by a stain circle pen and incubated in washing buffer I (1×PBS with 1% (v/v) Triton X-100) for an hour at room temperature. Then slides were incubated with the primary antibodies, including anti-γH2AX (raised in rabbit; Miao *et al.*, 2013), OsREC8 (raised in both rabbit and mouse; Shao *et al.*, 2011), OsMER11 (raised in mouse; Ji *et al.*, 2013), OsCOM1 (raised in mouse; Ji *et al.*, 2012), OsDMC1 (raised in mouse; Wang *et al.*, 2016), OsMER3 (raised in mouse; Wang *et al.*, 2009), OsPAIR2 (raised in mouse; Wang *et al.*, 2009) or anti-OsZEP1 (raised in mouse; Wang *et al.*, 2010) antibody solution (diluted 1:200 in blocking buffer: 1×PBS, 1 mM EDTA, 0.1% Tween 20, 5% BSA), at 4 ^o^C overnight. After three rounds of washing in washing buffer II (1×PBS with 0.1% (v/v) Tween 20), Alexa Fluor 488-conjugated goat anti-mouse secondary antibody or Alexa Fluor 555-conjugated donkey anti-rabbit secondary antibody (Beyotime, Shanghai, China) was added to the slides. The chromosomes were counterstained with DAPI (10 mg mL^–1^) in an anti-fade solution (Vector Laboratories).

## Results

### Identification and characterization of a sterile rice mutant

**Fig. 1.**
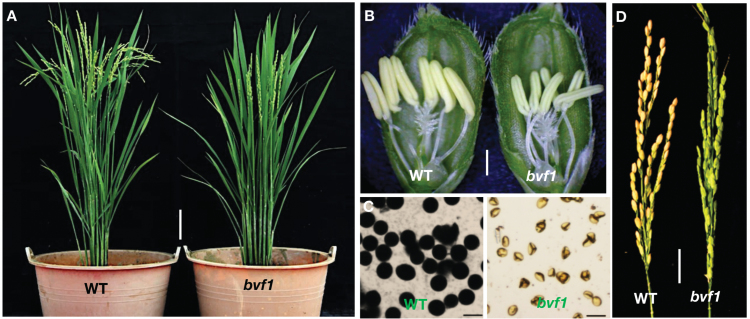
Phenotypes of the wild-type (WT; *japonica* cv Nipponbare) and the *bvf1* mutant. (A) The plants at heading and grain-filling stages. Bars, 10 cm. (B) Spikelets showing matured anthers. Bars, 1 mm. (C) Pollen grains stained with 1% I_2_–KI. Bars, 50 μm. (D) Spikelets after 4 weeks’ pollination. The sterile spikelets of *bvf1* were pollinated with WT pollen. Bars, 2 cm.

We created a mutant library of a *japonica* cultivar Nipponbare by ^60^Co γ-ray radiation. By screening the mutant library, we obtained a sterile mutant, named *bivalent formation 1* (*bvf1*) according to our later observation that the mutated causal gene affects bivalent formation in meiosis. The mutant had as normal vegetative growth as the WT plants, but with no seed setting at the reproductive growth stage (Fig. 1A, D). Further characterization showed that the mutant exhibited smaller anthers and completely sterile pollen grains (Fig. 1B, C). When the mutant plants were pollinated with WT pollen grains, no seed was produced, suggesting that the female gametes were also sterile. The segregation of fertile (104) to sterile (34) individuals in the progeny of self-fertilized mutant heterozygotes fitted the 3:1 ratio (Supplementary Table S2), indicating that a single recessive gene is responsible for the male and female sterile phenotypes.

### Meiosis is defective in pollen mother cells of *bvf1*

**Fig. 2.**
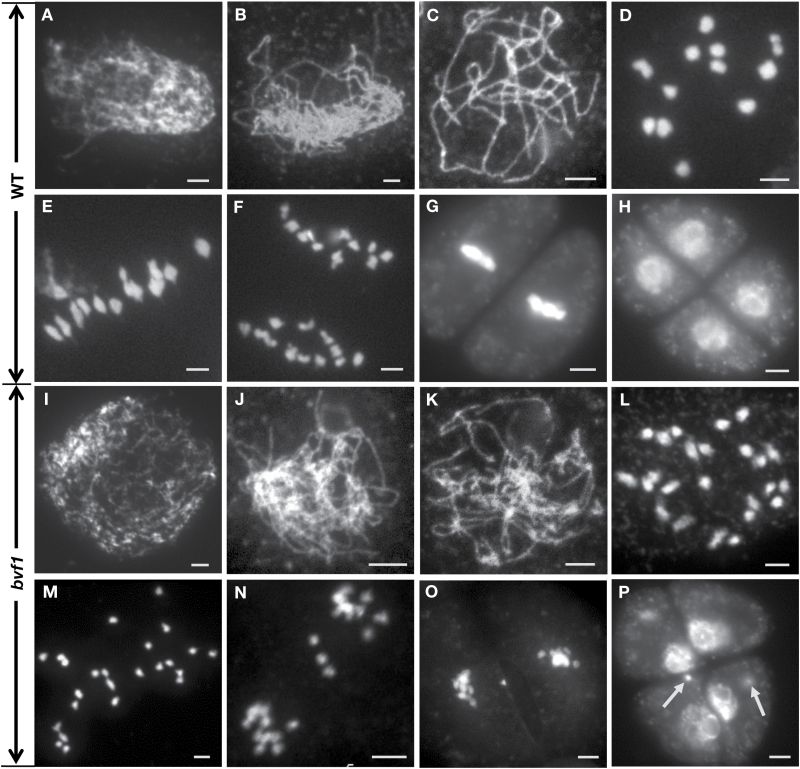
Observation of meiotic chromosome behavior in WT (A–H) and *bvf1* (I–P). The meiotic stages include leptotene (A, I), zygotene (B, J), pachytene (C, K), diakinesis (D, L), metaphase I (E, M), anaphase I (F, N), metaphase II (G, O), and tetrad (H, P). Arrows point to the micronuclei. Bars, 5 μm.

It is known that defective mutation of many meiotic genes causes male and female sterility in both human and plants (Székvölgyi and Nicolas, 2010; Luo *et al.*, 2014). To explore the possibility for the sterility in *bvf1*, we observed the meiotic chromosome behavior of pollen mother cells (meiocytes) using chromosome spreads stained with DAPI at different meiotic stages in both WT and *bvf1*. As shown in Fig. 2, in WT, at leptotene, the chromosomes began to condense and displayed a thread-like feature under microscopy (Fig. 2A). At zygotene, the homologous chromosomes aligned together and began to pair with each other (Fig. 2B). At pachytene, the homologs were stabilized by the synaptonemal complex (SC) and displayed thick thread-like chromosomes (Fig. 2C). At diakinesis, following the disassembly of the SC, the 12 pairs of homologs (also called bivalents) physically associated by chiasma and sister chromatid cohesion were observed (Fig. 2D). At metaphase I, all bivalents were aligned at the equatorial plate pulled by spindles (Fig. 2E), thereby resulting in the subsequent segregation to the opposite poles (Fig. 2F). Finally, the two dyads simultaneously underwent meiotic II cell division and formed the tetrad microspores (Fig. 2G, H).

Compared with the WT chromosome morphology, no obvious difference was observed in *bvf1* from leptotene to zygotene (Fig. 2I, J). At pachytene, unlike WT with fully synapsed homologs, the *bvf1* chromosomes condensed and aligned together, but did not show thick chromosomes (*n*=82 meiocytes) (Fig. 2K), suggesting a defect in synapsis. From diplotene to diakinesis, in contrast to the WT that formed 12 bivalents, *bvf1* had 24 univalents (*n*=110 meiocytes) (Fig. 2L), suggesting a failure of crossover formation. Due to the recombination defect, the 24 univalents were not well aligned at the equatorial plate at metaphase I (*n*=167 meiocytes) (Fig. 2M), and showed an uneven segregation to the two poles at anaphase I. Moreover, 70.9% *bvf1* meiocytes at anaphase I (*n*=79) had lagging chromosomes (Fig. 2N). At meiosis II, due to the unequal segregation of chromosomes, the *bvf1* meiocytes produced abnormal tetrads with uneven chromosome numbers and micronuclei (Fig. 2O, P). The failure of bivalent formation and the aberrant chromosome segregation provides an explanation for the complete sterility in *bvf1*. Together, these results indicate that *OsBVF1* is required for normal bivalent formation in rice meiosis.

### Map-based cloning of *OsBVF1*

**Fig. 3.**
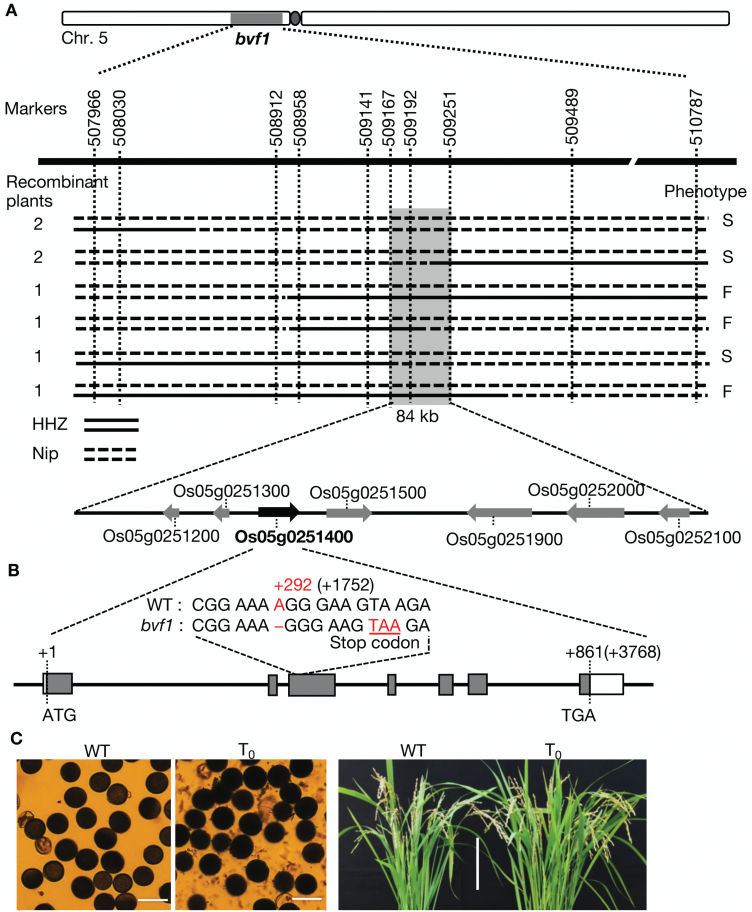
Map-based cloning of the gene for *bvf1*. (A) The Os*bvf1* locus was primarily mapped to a 2821-kb region on chromosome 5 using 775 segregation plants (F_2_ and BC_1_F_2_) of the *BVF1*/*bvf1*×HHZ (*indica* cv) cross, then further delimited to an 84-kb region by analysis of eight recombinant plants selected from the F_3_ and F_4_ populations. Seven genes have been predicted in this mapped region. (B) Sequencing analysis detected a single base (‘A’ nucleotide) deletion in the position +292 (+1752 including the introns) of the ORF of *Os05g0251400*. (C) The pollen (left) and grain-filled spikelet (right) fertilities of the transgenic plants (T_0_) with homozygous Os*bvf1* were restored by transformation with a binary construct expressing the ORF of *Os05g0251400*. Bars, 50 μm for pollen and 20 cm for plants.

To isolate the gene conferring the mutant phenotype, we crossed the heterozygous *BVF1*/*bvf1* plants (male and female fertile) with an *indica* rice variety, HHZ. The F_1_ plants were further backcrossed with HHZ. By linkage analysis using 10 sterile F_2_ plants and a set of polymorphic markers covering the whole genome, a region on the short arm of chromosome 5 was found to link with *bvf1.* Then we used a total of 775 F_2_ and BC_1_F_2_ plants and a number of molecular markers on this region (Supplementary Table S3) to primarily map the locus on a region of *ca* 2821 kb (Fig. 3A). Through further screening of new recombinants in the segregated F_3_ and F_4_ populations with the markers 507966 and 510787, *OsBVF1* (*Osbvf1*) was further delimited to an 84-kb region between two markers, 509167 and 509251 (Fig. 3A), a region that includes seven annotated genes. Then we amplified these genes by PCR for subsequent sequencing (Fig. 3A). A single base deletion in the third exon of the gene *Os05g0251400* was detected in *bvf1* (Fig. 3B), which caused a frame-shift and a premature stop codon (Fig. 3B). Because no other mutations in the other genes within the 84-kb region were found, we considered *Os05g0251400* as the candidate gene for *OsBVF1*.

To verify the function of *Os05g0251400*, we constructed a binary vector (pOX-BVF1) with the whole 1115-bp open reading frame sequence (AK103883) of *Os05g0251400* driven by the maize *ubiquitin* promoter. This construct was used to transform calli induced from immature seeds of the heterozygous *BVF1*/*bvf1* plants. By genotyping of the endogenous *Os05g0251400* in the transgenic-positive transgenic (T_0_) plants with the mutation site-specific primer set 1400-T (Supplementary Table S1), four out of 17 T_0_ plants were found to have homozygous *Osbvf1*, and they all showed recovered fertility and normal seed-setting (Fig. 3C and Supplementary Fig. S1). In the T_1_ generation of these four plants, the segregants with and without the transgenes co-segregated with the fertile and sterile phenotypes (Supplementary Table S4 and Supplementary Fig. S1). Therefore, we conclude that the single-base deletion in *Os05g0251400* is responsible for this sterile mutation of the target gene.

**Fig. 4.**
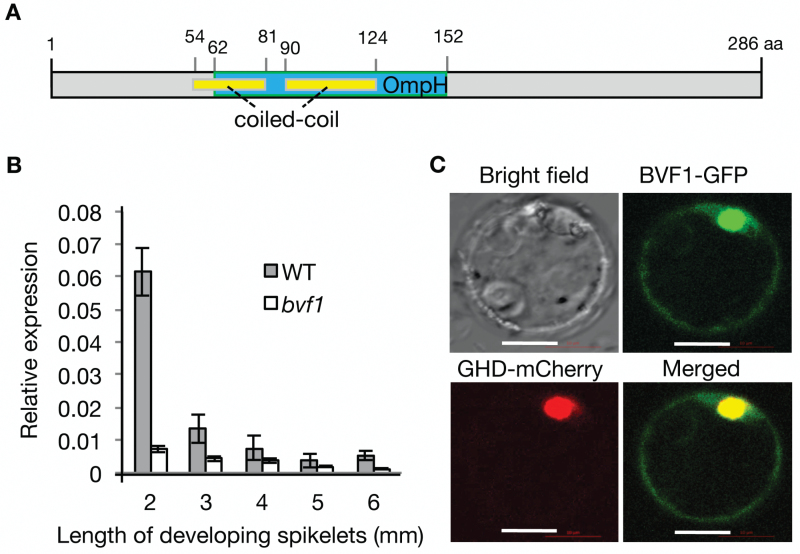
Structure of the OsBVF1 protein, the *OsBVF1* expression patterns, and the protein subcellular localization. (A) The structure of OsBVF1 with an OmpH domain and two coiled-coil motifs. (B) Expression patterns (mean with standard deviation of three biological replicates) of *OsBVF1* (WT) and *Osbvf1* in developmental spikelets. The spikelets of 2–3 mm in length were at the PMC to meiosis stages. *Actin 1* mRNA was used as the internal control. (C) The constructs expressing OsBVF1–GFP and a nuclear-localized fusion protein, GHD–mCherry, were co-transferred into rice protoplasts. Bars, 10 μm.

Sequence analysis (www.ncbi.nlm.nih.gov/, last accessed 13 March 2017) showed that *OsBVF1* encodes a hypothetical protein of 286 amino acids (aa) (protein Accession No.: NP_001055029) with a putative conserved OmpH (outer membrane protein H) domain from the 62nd to 152nd aa, and this protein is unique in the rice genome (Fig. 4A). The mutation in the *bvf1* allele produces a truncated protein of 99 aa. By running the ‘COILS’ program (http://www.ch.embnet.org/software/COILS_form.html, last accessed 13 March 2017) using OsBVF1 as query, it is predicted that OsBVF1 also can form two coiled-coil motifs in the central region (54–81 aa and 90–124 aa) (Supplementary Fig. S2), which partially overlaps with the OmpH domain (Fig. 4A and Supplementary Fig. S3). Thus, *OsBVF1* encodes a new protein with a unique OmpH domain coupling with the coiled-coil motif in rice.

### *OsBVF1* is highly expressed in anther and its protein targets to nucleus

To examine the expression profile of *OsBVF1*, we performed a qRT-PCR experiment and found that *OsBVF1* was expressed in various organs, with relatively higher level in anthers developing meiosis stages (Fig. 4B and Supplementary Fig. S4). We also found that the mRNA level was obviously lower in *bvf1* than in WT (Fig. 4B), probably due to degradation of the abnormal mutant mRNAs by the nonsense-mediated mRNA decay mechanism (Maquat, 2004). To investigate the subcellular localization of OsBVF1, we prepared a transient expression construct for an OsBVF1–GFP fusion protein. By co-transfer of the OsBVF1–GFP construct with a nucleus-localization marker construct expressing GHD–mCherry into rice protoplasts, we observed that the OsBVF1–GFP signal was mainly localized in nuclei, which overlapped with the GHD–mCherry signal (Fig. 4C), suggesting that OsBVF1 is a nucleus-localized protein.

### OsBVF1 is indispensable for meiotic DSB formation

**Fig. 5.**
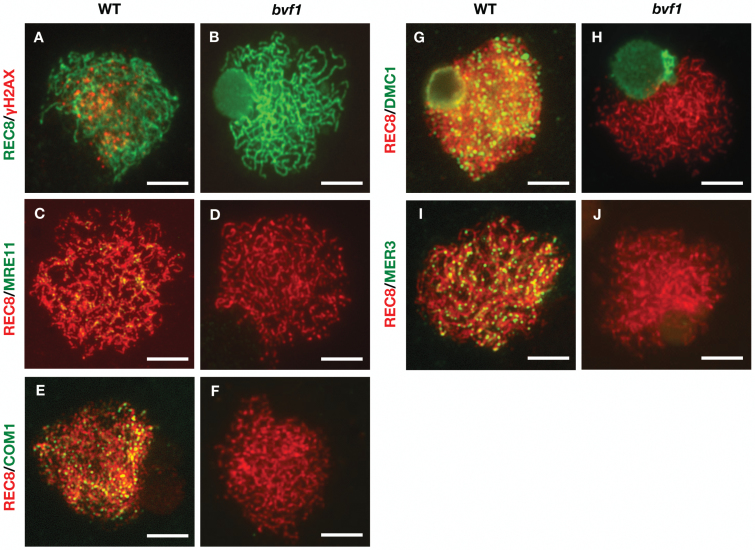
Dual immunostaining detection of several meiotic proteins in the WT and *bvf1* mutant. (A, B) OsREC8 (green) and γH2AX (red) signals at zygotene in WT (A) and *bvf1* (B). (C–J) OsMRE11 (green), OsCOM1 (green), OsDMC1 (green), OsMER3 (green) signals at zygotene in WT and *bvf1*. OsREC8 signals (red) were used to indicate the meiotic chromosome axes. Bars, 5 μm.

Meiotic recombination is initiated from the programmed DSB formation (Keeney *et al.*, 1997). The formation of DSBs triggers the phosphorylation of the histone variant H2AX (γ-H2AX), which specifically marks DSBs and facilitates post-replication DNA repair (Dickey *et al.*, 2009). To detect whether DSBs are formed in *bvf1*, we used immunofluorescence to examine the distribution of phosphorylated γ-H2AX with an anti-γH2AX antibody generated using the sequence from rice (Miao *et al.*, 2013). To mark the chromosomes, we used OsREC8, a homolog of Arabidopsis meiotic specific cohesin SYN1 (Cai *et al.*, 2003), which has a linear distribution pattern on chromosomes during early prophase I (Shao *et al.*, 2011). As shown in Fig. 5, WT zygotene meiocytes showed dot-like signals of γH2AX (Fig. 5A), while no signals were detected in *bvf1* (Fig. 5B), indicating that BVF1 is indispensable for rice meiotic DSB formation.

Following the DSB formation, the DSB ends are further processed by the MRX complex (Mre11/Rad50/Xrs2) and COM1/SAE2 (Mimitou and Symington, 2009). The rice OsMRE11 and OsCOM1 homologs have also been reported to participate in meiotic DSB repair (Ji *et al.*, 2012, 2013). We further examined the localization of OsMRE11 and OsCOM1 in both WT and *bvf1* mutant. Unlike WT with dot-like signals at pachytene chromosomes, we did not detect any signals of both proteins in *bvf1* at a similar stage (Fig. 5C–F), supporting the idea that OsBVF1 functions upstream of DSB end procession. This hypothesis was further supported by the undetectable signal of the other downstream proteins OsDMC1 (Wang *et al.*, 2016) and OsMER3 (Wang *et al.*, 2009) in *bvf1* mutant meiocytes (Fig. 5G–J). Taken together, these results provide strong evidence to support the role of OsBVF1 in DSB formation during meiotic recombination.

### OsBVF1 is dispensable for axial element installation, but required for the central element installation of SC

**Fig. 6.**
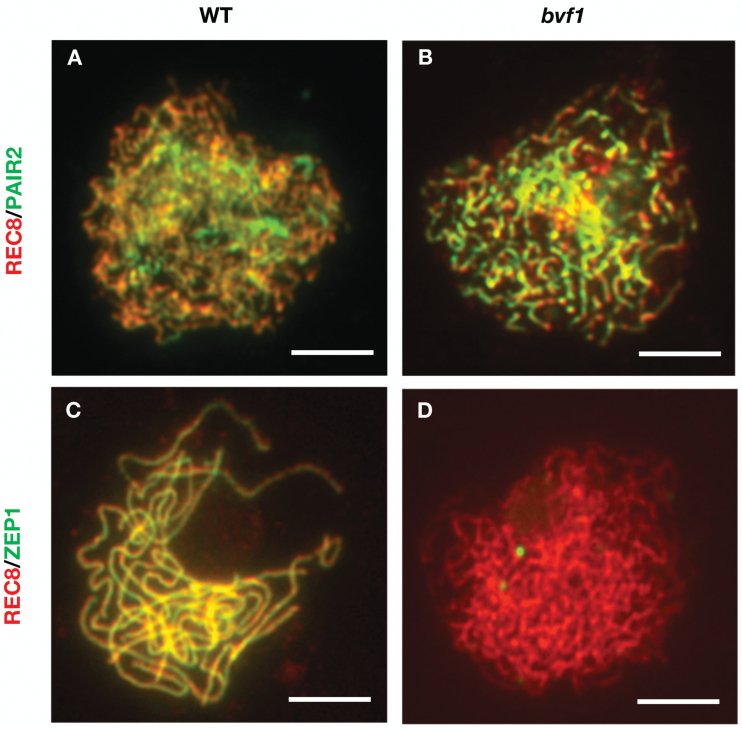
Dual immunostaining detection of two synaptonemal complex proteins in WT and *bvf1* mutant. (A, B) OsREC8 (red) and OsPAIR2 (green) signals at late zygotene in WT and *bvf1*. (C, D) OsREC8 (red) and OsZEP1 (green) signals at late zygotene in WT and *bvf1*. Bars, 5 μm.

After the progression of meiotic recombination, the SC, a proteinaceous structure including lateral and central elements formed between homologs, is important for the stabilization of recombination intermediates and facilitates subsequent homolog recombination (Zickler and Kleckner, 1999). The rice axial element (AE) protein OsPAIR2 is the homolog of yeast HOP1 and Arabidopsis AtASY1 (Nonomura *et al.*, 2007). We examined the localization patterns of OsPAIR2 in WT and *bvf1* meiocytes and found a normal linear pattern overlapping with zygotene chromosomes between WT and mutant (Fig. 6A, B), implying that the assembly of AEs is probably unaffected in the mutant.

To investigate whether the installation of SC occurs in *bvf1*, we examined the localization of rice OsZEP1 (Wang *et al.*, 2010), a homolog of Arabidopsis AtZYP1, the central element of the SC (Higgins *et al.*, 2005) in WT and mutant. The immunostaining signals for OsZEP1 at pachytene showed linear signals along with the entire chromosomes in WT meiocytes (Fig. 6C). By contrast, no such immunostaining signals were observed in the *bvf1* meiocytes (Fig. 6D). Thus, we conclude that OsBVF1 is required for the installation of the SC in rice, probably by an indirect effect due to lack of DSB formation in the mutant.

### OsBVF1 and its homologs are highly conserved in the plant kingdom

By protein homology search in NCBI (www.ncbi.nlm.nih.gov/, last accessed 13 March 2017) and other databases, we found a number of homologous proteins of OsBVF1 in different plant species. The homolog from wild rice, *Oryza brachyantha*, is 283 aa in length and shares the highest identity (89%) with OsBVF1. The homologs of other monocot plants have high levels of sequence identities to OsBVF1, such as *Brachypodium distachyon* (76.9%), *Sorghum bicolor* (75.1%), *Setaria italic* (80.2%), *Zea mays* (74.4%), *Hordeum vulgare* (73.1%), and *Triticum aestivum* (76.9%) (Supplementary Fig. S5). In contrast, the homologs from eudicots have relatively low levels of identities, such as *Arabidopsis thaliana* (41.5%), *Brasica rapa* (39.9%), *Carica papaya* (31.82%), and *Glycine max* (37.63%). Homologs of OsBVF1 also were found in the *streptophyta* plant *Klebsormidium flaccidum* (13.1%) and the alga *Coccomyxasu bellipsoidea* (14.2%). Moreover, nearly all the OsBVF1 homologs in the examined species can form one to two coiled-coil motifs except those from *Selaginella moellendorffii*, *Coccomyxa subellipsoidea* and *Klebsormidium flaccidum* (Supplementary Fig. S5).

**Fig. 7.**
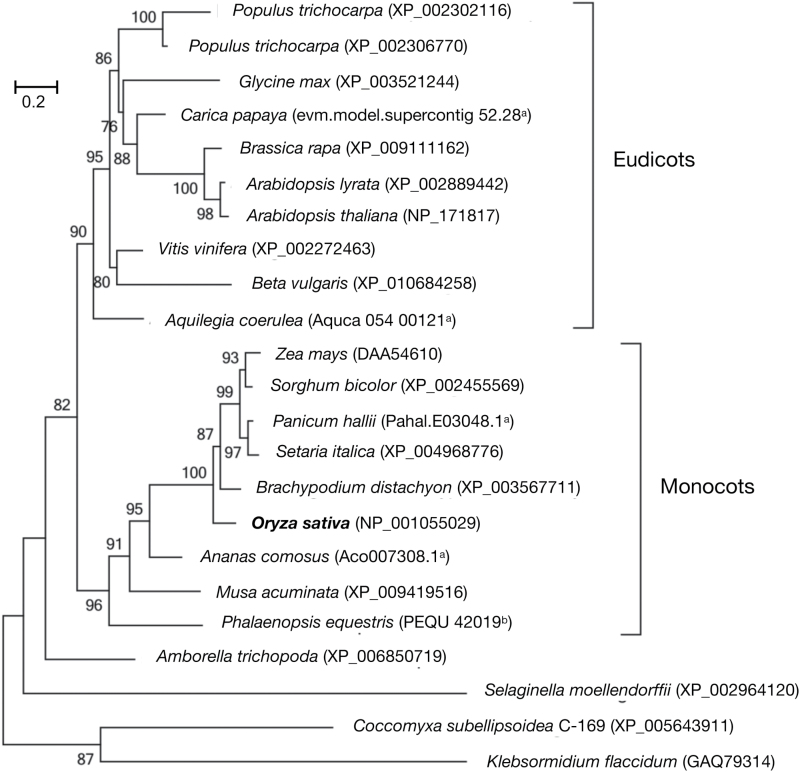
Phylogenetic analysis of plant proteins homologous to OsBVF1. The tree was constructed from the alignment of full-length proteins in rice and some representative plant species by MEGA6 using the neighbor-joining (NJ) model. The protein accession numbers are given in the brackets after species names. *The protein sequences are not available in GenBank, and are given in Supplementary Fig. S4.

According to the amino acid similarity, we built a phylogenetic tree among 23 representative plant species (Fig. 7). The proteins were divided into four groups among eudicots, monocots, pteridophytes and streptophyta/algae. The data suggest that OsBVF1 and the homologs are plant-conserved and they should be derived from a common ancestor. It is notable that, except for *Populus trichocarpa*, all the examined species have only a single copy of BVF1 or its homologs (Fig. 7), suggesting that the homologous genes did not expand during the evolution of plants.

## Discussion

### Identification of a new meiotic gene in rice

Most of the reported meiotic genes in plants are comparatively conserved from yeast to higher eukaryotes (Osman *et al.*, 2011; Luo *et al.*, 2014; Mercier *et al.*, 2015; Zickler and Kleckner, 2016). According to homology alignment in terms of sequence identity or similarity, previous studies have identified several meiotic genes in rice, such as *OsDMC1* (Ding *et al.*, 2001), *OsSPO11-4* (An *et al.*, 2011) and *OsRAD21-4* (Zhang *et al.*, 2006). Compared with yeast or fruit fly, plants with larger genome sizes are supposed to have more complicated meiotic regulation. As previously reported, some meiotic genes, such as *OsAM1* (Che *et al.*, 2011)/*AtSWI1* (Mercier *et al.*, 2001), *OsMOF1* (He *et al.*, 2016) and *OsPAIR1* (Nonomura *et al.*, 2004*a*)/*AtPRD3* (De Muyt *et al.*, 2009), were found to be plant specific, and the rice meiotic genes *OsMEL1* (Nonomura *et al.*, 2007) and *OsMEL2* (Nonomura *et al.*, 2011) have no homologs in other plant species. Therefore, meiotic control may vary somewhat among different species, even in plants. Thus, identification of more meiotic genes is necessary to expand our knowledge of meiosis. In this study, through mutant screening and map-based cloning, we identified *OsBVF1* from rice. Both sequence alignment and functional characterization support the fact that *OsBVF1* is a novel meiotic gene that encodes a coiled-coil motif- and OmpH domain-containing protein.

### The role of OsBVF1 in meiotic recombination

In this study, we provided several lines of evidence to support a role of OsBVF1 in rice meiosis. First, the rice OsBVF1 was required for fertility, and mutation of OsBVF1 caused male and female sterility; second, chromosome morphology analysis showed that *bvf1* was defective in formation of well-synapsed chromosomes and only produced univalents, suggesting a failure of synapsis and crossover formation; third, the meiotic recombination defect in *bvf1* is likely caused by failure of DSB formation, which is supported by the observation of the disappearance of the marker for localization of DSB and other proteins required for meiotic recombination; fourth, the undetectable OsZEP1 signal in *bvf1* suggests a role in SC formation, but the failure of the SC is likely a consequence of the initial defect in DSB formation (as reviewed in Gray and Cohen, 2016).

Sequence analysis showed that OsBVF1 has an OmpH domain and two coiled-coil motifs. It is reported that OmpH-containing proteins may play roles as protein folding catalysts or as chaperones in extracytoplasmic compartments (Missiakas *et al.*, 1996). In addition, the coiled-coil motifs play an important role in mediating subunit oligomerization in many proteins (Lupas, 1996; Mason and Arndt, 2004). Among the identified meiotic proteins, the central element of the SC shares a coiled-coil motif in the central region with one globular domain at each end, as with OsZEP1 in rice (Wang *et al.*, 2010), ZYP1 in maize (Golubovskaya *et al.*, 2011) and Arabidopsis (Higgins *et al.*, 2005), and Zip1 in budding yeast (Sym *et al.*, 1993; Page and Hawley, 2004). The coiled-coil motifs of the proteins form ladder-like or hinge-like parallel structures in the central region of the SC. Besides, some other meiotic proteins also contain the coiled-coil motifs, including OsPAIR1, OsPAIR3, OsAM1, OsSGO1, and OsHEI10 in rice (Nonomura *et al.*, 2004*a*; Che *et al.*, 2011; Wang *et al.*, 2011*a*, 2012*a*; Wang *et al.*, 2011*b*), DSY2 in maize (Lee *et al.*, 2015) and RED1 in budding yeast (Smith and Roeder, 1997), providing evidence that the coiled-coil motif is one of the important domains among proteins required for meiosis. Since our yeast two-hybrid assay did not detect any interaction between OsBVF1 and OsPAIR2/3 (Nonomura *et al.*, 2007; Yuan *et al.*, 2009; Wang *et al.*, 2011*a*), OsZEP1 (Wang *et al.*, 2010) and OsCRC1 (Miao *et al.*, 2013), it is likely that OsBVF1 may not directly participate in SC assembly in rice.

### The function of OsBVF1 and its homologs might be highly conserved in plants

Research into evolutionary biology has indicated that between 55 and 75 million years ago, plants had their genomes duplicated so as to increase the chance of survival (Lohaus and Van de Peer, 2016). Comparison of plant species showed that at least several million years ago, many monocot lineages, including wild rice, had already experienced two distinct paleopolyploidies (Jiao *et al.*, 2014). Lots of genomic hints can verify this notion, for example, the highly conserved meiotic genes such as *OsDMC1* (Ding *et al.*, 2001; Metkar *et al.*, 2004), *OsRAD51* (Rajanikant *et al.*, 2008) and *OsPAIR2* (Nonomura *et al.*, 2004*b*) all have two copies in the rice genome, with one of the copies silenced like *OsPAIR2* or both functionally reserved. Interestingly, in the case of OsBVF1 and its homologs (orthologs), from algae to monocots and eudicots, only a single copy of the gene was reserved in all the genomes (except for *P. trichocarpa*) during plant genome evolution, suggesting that these orthologous genes may be important for plant sexual reproduction. Therefore, we infer that OsBVF1 and its homologs in other plants may be highly conserved, with a primary role in reproduction. Both the present results and previous findings indicate that some plant-specific genes, including *OsBVF1*, have evolved in the regulation of plant-specific meiosis.

## Supplementary data

Supplementary data are available at *JXB* online.

Fig. S1. Genetic and phenotypic analyses of the *OsBVF1*-transgenic plants.

Fig. S2. Coiled-coil motif prediction of OsBVF1 and OsPAIR3 based on the web-tool COILS.

Fig. S3. Comparison of OmpH and coiled-coil motif sequences of OsBVF1 and OsPAIR3.

Fig. S4. Expression pattern of *OsBVF1* according to the Rice Expression Profile Database.

Fig. S5. Sequence alignment of OsBVF1 and its homologous proteins.

Table S1. Primers used in the study.

Table S2. Segregation of fertile and sterile plants in *bvf1* M_3_ lines.

Table S3. Segregation of fertile and sterile plants in *bvf1* mapping populations.

Table S4. Sequences of OsBVF1 homologs of some plant species that are not available at GenBank.

## Supplementary Material

Supplementary_Table_S1_S4_Figures_S1_S5
