# Supplementary material for: Bivalent Formation 1, a plant-conserved gene, encodes an OmpH/coiled-coil motif-containing protein required for meiotic recombination in rice
Source: J Exp Bot. 2017 Mar 24;68(9):2163–74. doi: 10.1093/jxb/erx077 (PMC5447885; doi:10.1093/jxb/erx077)
Supplement: Supplementary_Table_S1_S4_Figures_S1_S5 [file erx077_suppl_Supplementary_Table_S1_S4_Figures_S1_S5.pdf]

Supplementary Tables

Table S1. Primers used in the study

**Name**	**Forward (5’ to 3’)**	**Reverse (5’ to 3’)**
**For mapping**		
507966	GCATGAAGCAATCCTGGATCATC	GCTGCTGGTATTAAATATATATTGCAAG
508030	ACGAGTCATCCGCAAACAG	GTGCAGAGGTGTTCTCGTC
508912	CGGAGAAGTGTTGGCCATA	GATCGGTCACTGTTGACTATTGA
508958	CAACTAGAGAGGTAGGCA	GCTCTCGAACCTATTTCGA
509141	CGTTCAGCCAGATCGAACA	GCAAAAGTTGCTCTGAACTCTCT
509167	GTGCCGAAGAAAGCGAAGA	GGAGGAAGTGCCTTGGTA
509192	CGTTCAGCCAACGTTTAGCA	GTGCTGCAATCACAAACTCCA
509251	GTTGGTCGATCAGTTATGTTGCA	GTTCCGGCTAGCTAGTTTGT
509489	GGCTTACTTATAGATGTTAATAGTAG	GCATAGGCATGTAACTGTTATAAG
510787	GAGGCGTAAAGGTTTTGCATG	CAGCCTCATTTCAGTCAACAAG
**For vectors construction**		
pOX-BVF1	GAACGGTACCCGGCGGAAGGATGG<br>AGA	AAACGGATCCTAAGTAAAATGGTGTG<br>GAGATTC
BVF1-GFP	*TCAAGCTTCGAATTCTGCAGT*<br>ATGGAGAGGGCTACCACCTCC	*CCCGGGCCCGCGGTACCGTCG*<br>CTCTTCTAATGACGCCTTGCA
GFP-BVF1	*CGAGCTCAAGCTTCGAATTCT*<br>ATGGAGAGGGCTACCACCTCC	*GCCCGCGGTACCGTCGACTGC*<br>TCACTCTTCTAATGACGCCTT
**For transformant analysis**		
HPT	ATTTGTGTACGCCCGACAGT	GTGCTTGACATTGGGGAGTT
pOX-T	TTGTCGATGCTCACCCTGTTG	ACCGGCAACAGGATTCAATC
BVF1-T	GTCACCATTAGCATCTACGC(F)	GCAAGATTCTCCTTACACTG (R1)<br>CCGTCTTACTTCCCGTTTC (R2)

Note: underlined characters indicate the restriction sites, italic characters indicate the vector sequences.

Table S2. Segregation of fertile and sterile plants in *bvf1* M_2 and M_3 lines

Lines	Fertile plants	Sterile plants	Total plant	χ^2 (3：1)
M_2-8	28	10	38	0.035
M_3-8-1	16	5	21	0.016
M_3-8-2	16	3	19	0.860
M_3-8-4	14	3	17	0.490
M_3-8-5	16	6	22	0.061
M_3-8-12	14	7	21	0.778
Sum	104	34	138	0.010

Note: f = 1, χ^2 (3:1) < 3.84, $P > 0.05$.

Table S3. Segregation of fertile and sterile plants in *bvf1* mapping populations

**Lines**	**Fertile plants**	**Sterile plants**	**Total plant**	χ^2 **(3:1)**
8-6 (F_2)	64	24	88	0.242
8-7-1(F_2)	104	29	133	0.724
8-7-2(F_2)	112	22	134	5.264
8-7-4(F_2)	106	51	157	4.690
8-7-5(F_2)	113	35	148	0.144
8-6BC_1F_2	78	37	115	3.157
Sum	577	198	775	0.124

Note: f = 1, χ^2 (3:1) < 3.84, $P > 0.05$; χ^2 (3:1) < 6.63, $P > 0.01$.

Table S4. Sequences of BVF1 homologs of some plant species that are not available in the GenBank.

Species	Sequence
Caraca papaya<br>(evm.model.supercontig 52.28*)	MREIKREIKRVEKLMNTVSSLQTALQLMVREIPNVQKVMLVLGGSPIR<br>PRYVYELFFSHGEVVSSGSGDFSKSKAAEMLSRKAIRALISKDAGSSS<br>YPGPTKLFLLVKAPSSFNLPQHFLPKRDFRYSKKIVPFRLRFKCRTRVG<br>EMDSLHHASQTRNSTCLKDAISNDFIWFQCRHVIKGLALEGREEE
Aquilegia coerulea<br>(Aquca054 00121*)	MRRREEGEGEGESRSESENYIKEIETTAETLDEAVIFHVIKEIVGFVLY<br>MHQQIPSLLQDLNQEFDELQTGRKDLELVLTQTEVKASSRRKHIGRM<br>REVKQGIKRLEKLMSSVSVLQTAIQLMLHEIHSIQGVMLVLGSSPVRP<br>QHVYEMLFSHGRVVSDHGKENTKSKVAEALSRKAIRALISSGAGSVS<br>YAGPTKLFIMVKAPATLNLPLHFLPKRDFRYSKKIVPFKLRIKCKTRYH<br>DMGTSHHCPPPSIALPGATSNDLIWFQCRHAIKGLTIKTPLTEE
Panicum hallii<br>(Pahal.E03048.1*)	METNTSSSSSGGGGAGAGTEQRGLGLVEVQAAAAALRRSEVFHVVK<br>ELVGFVLYMHHQIPSVLQSLENEFASLKEEMTEMTLQPAELKPSDQIK<br>YNTRKREVRCRIKKHEKLMNGISTLLCALQQALDEVSSIEGVVLILGG<br>SLVRPLFVYDITISHGTFDSGSAKDQALTKLAQSVSRKAIRALVSCGAG<br>SLSYTGPSKLFLLVRCPSTLNLPLDFLPKRDFRYSKKVVPLQMHIKCSK<br>AGCQWNNQHHMPIANAPCSTSESSPSDAIWFQCKHTIRGLPGKASLE<br>G
Ananas comosus<br>(Aco007308.1*)	MESTDEGGGGGELGLGLGFAEIGAVAEGLGRSEVFHIVKEVIGFVLYM<br>HHQIPSVLQSLENEFVALKEEFKNLVDSGTMPAETKASDQRKHNMRK<br>REVKQGIKRQEKLMNGISSLLSALQDALDELPSIQGVTLVLGGSLARP<br>LHVCDMLFSNGRFDSGSAKECTKSKVAQTLSRKAIRALISSGAGSTST<br>GPCKLFLLMKCPCTFNLPLHFSPKREFHYTKKVVPFRLHIKCKAKDK<br>AMNDQHSNTISSSCCMSESAQTEDI
Phalaenopsis equestris<br>(PEQU 42019*)	MLSFFRVLQHLEHEFNGLKEDYKQLELLHSTGVERKEKNIYFHRKAN<br>MRKNEVKSSIKKLEKLMSSISTLFSALQEAIDGIPDFHGVTFLLGGSPA<br>RPQHVYEIFFSHFGVNSDNVNHITKSKVAEALSRKVVRALMSNDAGS<br>SISGPTKLFLMIKCPCTFNLPLHFLPKRDFRCNKKVLPFKLHIKSKMGD<br>QIMKDLHYNSGNTNSSSLADMVANDMIWYQCRHAVKGLAFKSHETD<br>C

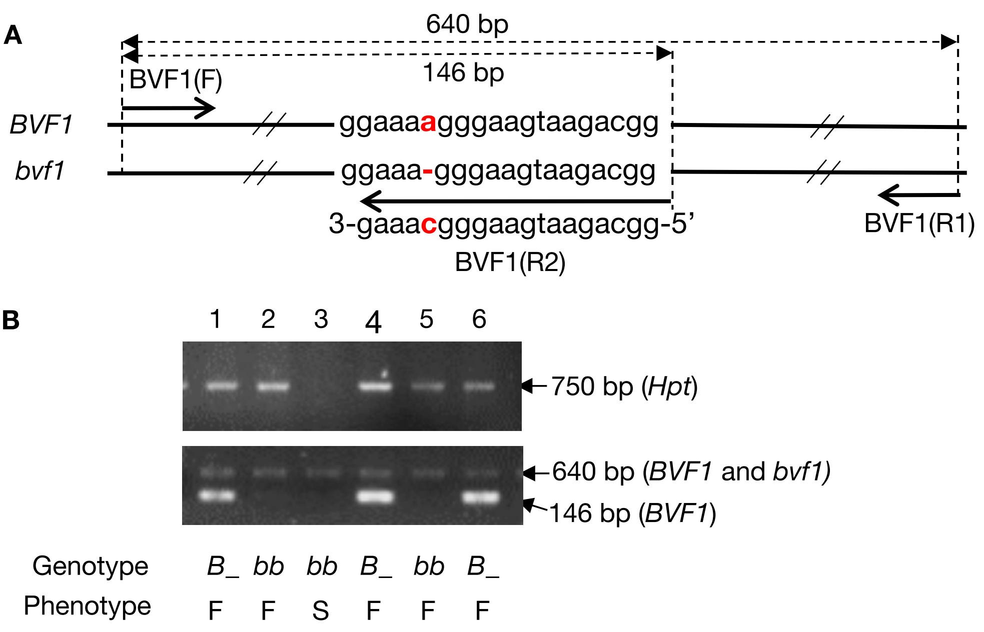

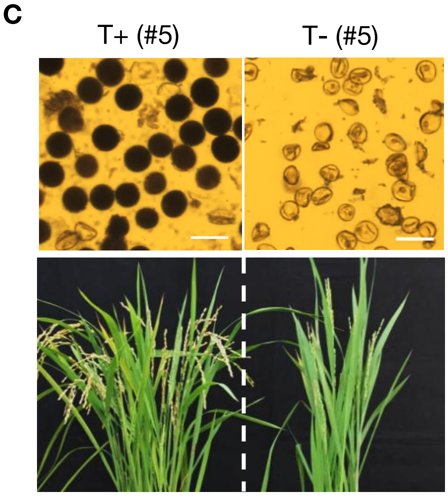

D

T1 family	T^+, phenotype	T^-, phenotype
#2	35, F	7, S
#5	15, F	9, S
#10	17, F	5, S
#15	8, F	4, S

Fig. S1. Genetic and phenotypic analyses of the *OsBVF1*-transgenic plants. (**A**) Illumination of a PCR strategy using three primers for genotyping the T_0 plants. BVF1(F) and BVF1(R) are located on the second and third intron, thus could amplify only the endogenous *OsBVF1* and *Osbvf1* alleles but not the cDNA *OsBVF1*-transgene. The 146-bp fragment was specifically amplified from *BVF*1 by the *OsBVF*1-specific primer BVF1(R2) combining with BVF1(F). (**B**) Analysis of the T_0 plants by PCR using *Hpt*-specific primers (*Hpt,* indicating the presence of the T-DNA with the cDNA *OsBVF1*-transgene) and the primers shown in (**A**). Plant #3 was transgenic-negative, and the transgenic-positive plants #2 and #5 had homozygous *Osbvf1* (*bb,* without the *OsBVF1*-specific 146-bp band) with the fertility recovered by the transgene. *B*_ indicates homozygous *OsBVF1* or heterozyote. F, fertile. S, sterile. Only results for six of the 17 T_0 plants are shown. (**C**) The morphologies (grain-filling stage) and pollen phenotypes of the T_1 segregant plants with (T+) and without (T-) the transgene derived from a T_0 (#5) plant. Bar = 50 µm. (**D**) Co-segregation of the transgene (T+) with the recovered fertility in the T_1 families.

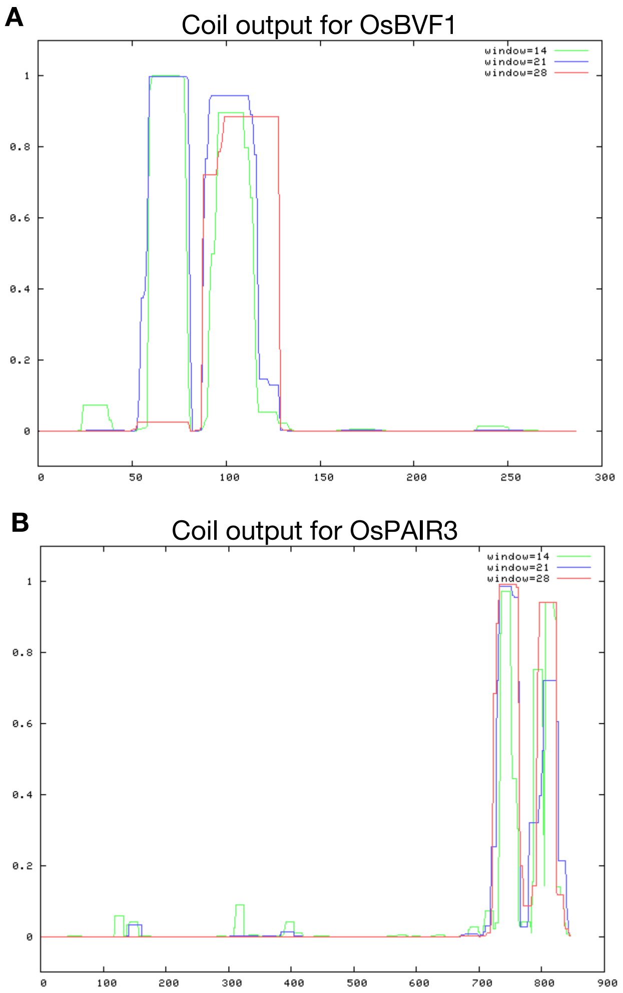

Fig S2. Coiled-coil motif prediction of OsBVF1 (**A**) and OsPAIR3 (**B**) based on the web-tool COILS (http://www.ch.embnet.org/software/COILS_form.html).

```
OsBVF1   54  MHHQIPAVLQNLENEFAS.LKEEMTEMALPPGEMKPSDQRKYNTRKREVRRRIKKQEKLMN  112
OsPAIR3 723  DVDKLVNAGKSKRKRLESTFEEQQEKLRILHEKFKEEVNQQLLGCKNSVED.FEAYHAELK  782

OsBVF1  113  GLSS.VFSALQKALDEVPSIEGVLLLLGGSLVRPLFVYDITISHGRFDAG  161
OsPAIR3 783  GVADKQKASHKKLLQNAEKTVGAQLSDAETKIAEVQKRARKRMKGLKFVL  832
```

Fig. S3. Comparison of the OmpH/coiled-coil motif sequences of OsBVF1 and OsPAIR3. No significant similarity is present between the sequences. The OmpH domains are shown with an up line or an under line, and the coiled-coil motifs with boxes .

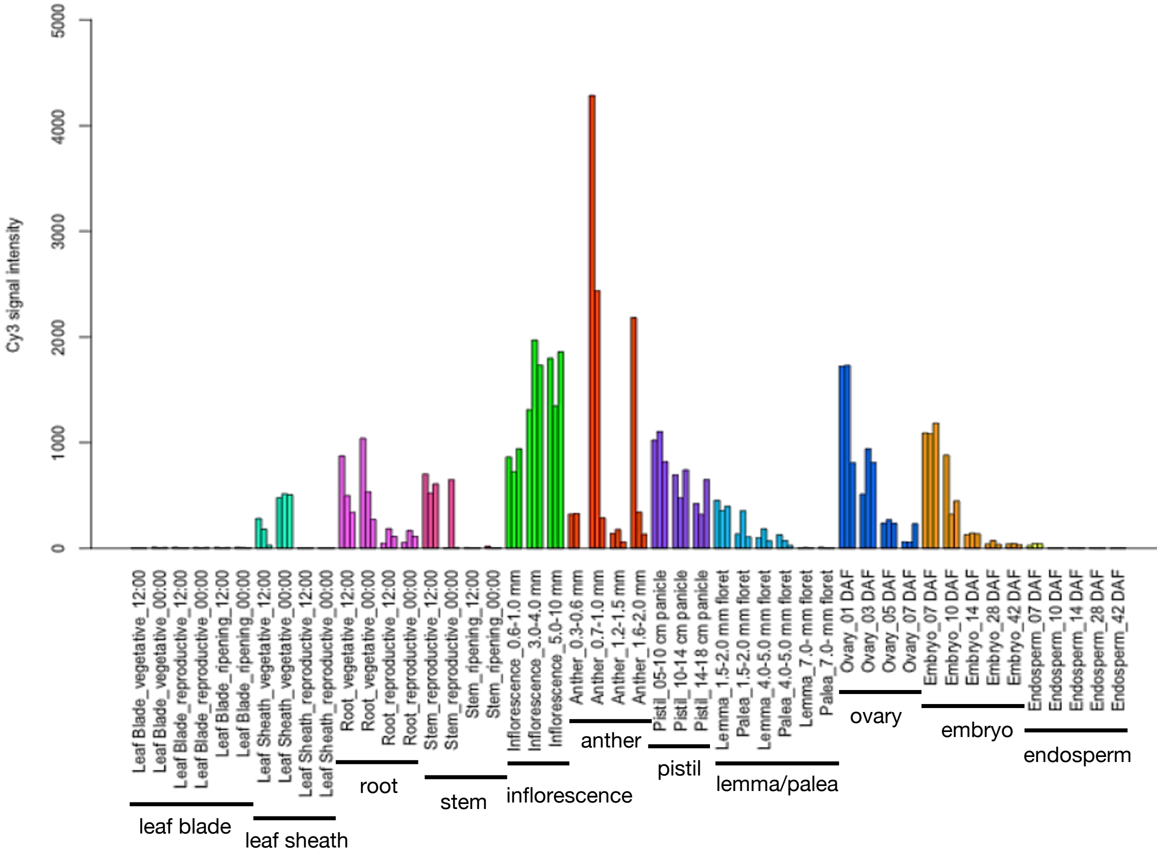

Fig. S4. Expression pattern of *OsBVF1* (*Os05g0251400)* according to the Rice Expression Profile Database (RiceXPro, http://ricexpro.dna.affrc.go.jp/). *OsBVF1 was* expressed in many vegetative and reproductive tissues except for leaf blade, leaf sheath of reproductive-stage, stem of ripening-stage and glume, and endosperm. The anthers of 0.3-0.6 mm are at sporogenous cell to PMC stages, anthers of 0.7-1.0 mm are at meiosis to early-mononucleate stage, anthers of 1.2-1.5 mm are at binucleate stage, anthers of 1.6-2.0 mm are at tricellular (mature) pollen stage.

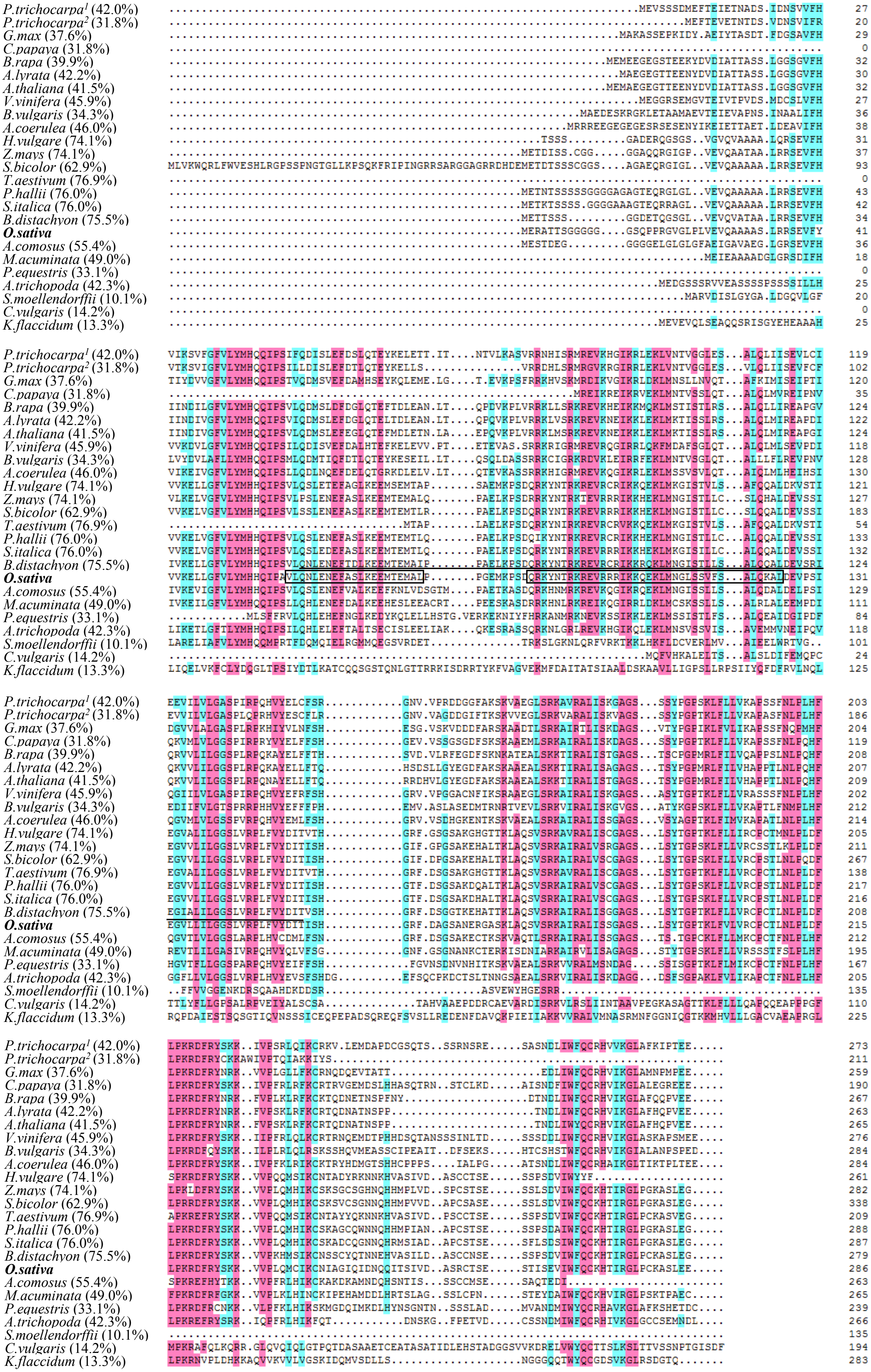
P.trichocarpa1 (42.0%)
P.trichocarpa2 (31.8%)
G.max (37.6%)
C.papaya (31.8%)
B.rapa (39.9%)
A.lyrata (42.2%)
A.thaliana (41.5%)
V.vinifera (45.9%)
B.vulgaris (34.3%)
A.coerulea (46.0%)
H.vulgare (74.1%)
Z.mays (74.1%)
S.bicolor (62.9%)
T.aestivum (76.9%)
P.hallii (76.0%)
S.italica (76.0%)
B.distachyon (75.5%)
O.sativa
A.comosus (55.4%)
M.acuminata (49.0%)
P.equestris (33.1%)
A.trichopoda (42.3%)
S.moellendorffii (10.1%)
C.vulgaris (14.2%)
K.flaccidum (13.3%)

Fig. S5. Sequence alignment of *O. sativa* OsBVF1 protein (Accession No. NP_001055029) with the homologous proteins of *Populus trichocarpa*[1] (XP_002306770, 42.0%), *Populus trichocarpa*[2] (XP_002302116, 31.8%), *Glycine max* (XP_003521244, 37.6%), *Carica papaya* (evm. model. supercontig 52.28, 31.8%), *Brassica rapa* (XP_009111162, 39.9%), *Arabidopsis lyrata* (XP_002889442, 42.2%), *Arabidopsis thaliana* (NP_171817, 41.5%), *Vitis vinifera* (XP_002272463, 45.9%), *Beta vulgaris* (XP_010684258, 34.3%), *Aquilegia coerulea* (Aquca 054 00121, 46.0%), *Zea mays* (DAA54610, 74.1%), *Sorghum bicolor* (XP_002455569, 62.9%), *Panicum hallii* (Pahal.E03048.1, 76.0%), *Setaria italica* (XP_004968776, 76.0%), *Brachypodium distachyon* (XP_003567711, 75.5%), *Ananas comosus* (Aco007308.1, 55.4%), *Musa acuminata* (XP_009419516, 49.0%), *Phalaenopsis equestris* (PEQU 42019, 33.1%), *Amborella trichopoda* (XP_006850719, 42.3%), *Selaginella moellendorffii* (XP_002964120, 10.1%), *Coccomyxa subellipsoidea* (XP_005643911, 14.2%), *Klebsormidium flaccidum* (GAQ79314, 13.1%). Identities with OsBVF1 were given after accession numbers. Similarities of the sequences showed in different colors, dark 100%, pink 75% blue, 50%. The OmpH domain of OsBVF1 is marked by an up line and the coiled-coil motifs are boxed.
